# Comparison of Outcomes Between Anatomical and Traditional Lung Volume Reduction Surgery for Severe Emphysema

**DOI:** 10.3390/jcm15031121

**Published:** 2026-01-31

**Authors:** Ra’fat Tawalbeh, William Ansley, Paula Browne, Rachel Braithwate, Hilmar Spohr, Akesh Dhrampal, Sadiyah Hand, Malcolm Marquette, Vasileios Kouritas

**Affiliations:** 1 Department of Thoracic Surgery, Norfolk and Norwich University Hospital, Norwich NR4 7UY, UKwilliam.ansley@nnuh.nhs.uk (W.A.); 2 Oxygen and Chronic Obstructive Pulmonary Disease Services, Norfolk and Norwich University Hospital, Norwich NR4 7UY, UK; paula.browne@nnuh.nhs.uk (P.B.); rachel.braithwaite2@nnuh.nhs.uk (R.B.); 3 Department of Radiology, Norfolk and Norwich University Hospital, Norwich NR4 7UY, UK; hilmar.spohr@nnuh.nhs.uk; 4 Department of Anesthesia and Critical Care, Norfolk and Norwich University Hospital, Norwich NR4 7UY, UK; akesh.dhrampal@nnuh.nhs.uk; 5 Department of Respiratory Medicine, Norfolk and Norwich University Hospital, Norwich NR4 7UY, UK; sadiyah.hand@nnuh.nhs.uk (S.H.); malcolm.marquette@nnuh.nhs.uk (M.M.)

**Keywords:** emphysema, lung volume reduction surgery, anatomical resection, thoracic surgery

## Abstract

**Background:** Anatomical lung resection is performed in cancer patients with severe emphysema who may also benefit from lung volume reduction (LVR). However, anatomical lung volume reduction surgery (LVRS) for emphysema alone is uncommon. This study compares the outcomes of anatomical and traditional LVRS. **Methods:** Patients undergoing surgery for emphysema were retrospectively analysed. They were grouped as anatomical LVRS (A-LVRS) and traditional LVRS (T-LVRS) patients. Various outcomes were compared between groups. **Results:** Thirty-three (33) patients were divided into A-LVRS (14) and T-LVRS (19) groups. The mean age was 62.1 ± 8.8 and 17 (51.5%) were females. Demographic and preoperative variables were similar between these groups. Overall complications, length of stay (LOS), critical care complex (CCC) re-admission, CCC-LOS and hospital re-admissions were similar. Drain stay duration was shorter in the A-LVRS vs. the T-LVRS group (6.4 vs. 12.6 days, respectively, *p* = 0.042) and air leak-related complications were also fewer in the A-LVRS group (21.4% vs. 57.9%, respectively, *p* = 0.036). Reduction in the COPD assessment test was greater in the A-LVRS vs. T-LVRS group (17 vs. 7.8, *p* = 0.045). Forced expiratory volume 1 s (FEV_1_) was improved by 8.25% in the A-LVRS vs. 2.9% in the T-LVRS group (*p* = 0.049). The lung transfer factor for carbon monoxide (TLCO) increased by 7.9% in the A-LVRS group versus a decrease of −1.01% in the T-LVRS group (*p* = 0.031). More lung volume was removed in the A-LVRS vs. the T-LVRS group (1625.4 vs. 352.4 cm^3^, *p* = 0.035). In-hospital/30-day/90-day deaths and long-term survival were similar. **Conclusions:** Anatomical LVRS is safe and may provide better outcomes in selected parameters compared with traditional LVRS.

## 1. Introduction

Lung volume reduction surgery (LVRS) has evolved since the initial criteria were established, according to which LVRS could be offered only in specific and highly selected patients [1,2]. Nowadays, LVRS can be performed in the form of lobectomies, mainly in cancer patients who are also found to have severe emphysema and who could benefit from the lung volume reduction (LVR) effect [3,4,5].

Traditional LVRS, in the form of a wedge resection of the emphysematous lung part, has been shown to improve respiratory function as well as cardiopulmonary circulation [6,7]. Clinical improvement was shown to be more profound in emphysema patients subjected to lobectomy [8]. However, it is not entirely certain if this is indeed true, because these effects were variable in cancer and thoracotomy patients and the comparison involved different technical considerations [9].

In this study, we aimed to compare outcomes between severe emphysema patients who underwent sublobar or lobar anatomical LVRS with those who underwent traditional LVRS in the classic form of a long wedge resection.

## 2. Patients and Methods

### 2.1. Study Design

Severe emphysema patients discussed at our institute’s emphysema MDT with a decision to undergo LVRS between June 2021 and January 2025 were included in this study. Patients were divided into 2 groups: a group of patients who underwent anatomical LVRS in the form of targeted segmentectomy, bisegmentectomy, trisegmentectomy, basal segmentectomy or lobectomy (A-LVRS) and a group of patients who underwent traditional LVRS in the form of a long wedge resection of emphysematous lung parenchyma (T-LVRS).

Multiple preoperative, intraoperative and postoperative outcomes were retrospectively compared between these 2 groups.

### 2.2. Emphysema MDT and Patient Selection

All patients included in this study were treated via our institution’s emphysema program. All patients underwent a similar investigation pathway including a 1 mm chest CT followed by Strat-X™ analysis (Pulmon-X Corp, Redwood City, CA, USA), a perfusion scan, an echocardiogram (including right heart measurements for pulmonary hypertension probability) and full lung function tests (LFTs), including static volumes. All patients were also seen at our service’s hyperinflation clinic, where they had a cotinine test (to verify smoking cessation), a 6-minute walking test (on O_2_ if applicable), calculation of body mass index, airflow obstruction, dyspnea and exercise capacity (BODE) index and Borg scale, and measurement of arterial blood gasses. Moreover, the COPD assessment test score (CAT score) and the modified Medical Research Council (mMRC) dyspnea score were determined. All patients discussed had stopped smoking for at least 6 months (including vaping) and had at least 1 rehabilitation program completed. All patients were finally discussed at the hyperinflation–emphysema multidisciplinary team meeting (MDT) of our service. Patients who were not candidates for endobronchial valve insertion, as per the MDT decision, or wished to have surgery, were referred for LVRS. Patients who were found to have proven pulmonary hypertension were not included in this study.

Based on imaging showing better quality lung parenchyma in the target lobe, and in order to resect the emphysematous area as completely as possible, a decision was made in some cases to proceed to a sublobar or a lobar anatomical resection (i.e., in the scenario of a totally destroyed target lobe) rather than a traditional long wedge-form LVRS deep into the diseased parenchyma. Buttressed reinforced staplers were variably used. All procedures were conducted via a minimally invasive approach; i.e., either via VATS (video-assisted thoracoscopic surgery with a utility incision and 1 or 2 accessory ports) or RATS (robotic-assisted thoracoscopic surgery with 4 ports utilized: 1 × 12 mm and 3 × 8 mm ports and no assistant port).

All procedures included in this study were unilateral and performed by a single surgeon.

### 2.3. Data

Apart from patient demographics (age and gender), other data investigated included LFTs pre- and 6–12 months post-intervention (forced expiratory volume in 1 sec—FEV_1_, total lung transfer factor for carbon monoxide—TLCO and residual volume—RV), patient co-morbidities, pre- and post-interventional data (including CAT scores, mMRC dyspnea scores and 6MWTs), interventional data (including the surgical approach (RATS or VATS) and type of A-LVRS (e.g., segmentectomy, bisegmentectomy, lobectomy, etc.)), the side on which surgery was performed, upper/lower lobe procedures, conversions, the duration of the procedure and the sequelae during surgery. Postoperative data included patients with all/any complications such as air leak-related complications (e.g., a prolonged air leak > 5 days, discharge with a flutter bag, re-interventions for air leaks such as skin incisions for subcutaneous emphysema, re-insertion of drain, blood patch, etc.), respiratory complications (including exacerbation of COPD, chest infection, etc.), returns to the theatre, the volume of the specimen removed, re-admissions, subjective breathing changes as reported by patients, overall length of stay (LOS) for this admission, planned and unplanned postoperative admission to the critical care complex and CCC-LOS, the drain length of stay (DLS), in-hospital/30-day deaths and 90-day deaths) and survival until the end of this study.

### 2.4. Follow-Up

According to our institute’s emphysema service pathway, all patients were seen by the surgeon 4–6 weeks post-surgery to ensure that there were no issues from the procedure. The next follow-up review was 3 months post-intervention over the phone and then 6–12 months post-intervention at our institute’s hyperinflation clinic, where post-intervention mMRC dyspnea scores, CAT scores, LFTs, 6MWTs and the rest of the scores were determined. All patients were followed up further under the care of the surgeon.

### 2.5. Analysis

Statistical analysis was performed with IBM SPSS Statistics for Macintosh, Version 29.0, Armonk, NY, USA: IBM Corp. Quantitative data are presented as mean ± standard deviation (SD) and were investigated for normality of distribution with the Shapiro–Wilk’s test (normally distributed when *p* > 0.05) and Q–Q plots (acceptable figures for normality of distribution). Categorical data are presented as the number of observations and percentages.

There were no missing entries in all variables as the emphysema support group ensured adequate capture and scrutinisation of all data.

Statistical significance was determined with the Student’s *t*-test, Mann–Whitney (when Levene’s test was *p* < 0.05 and not normally distributed according to the previous normality testing), chi-square and Fisher’s exact tests. The statistical significance level was set to a *p* value < 0.05.

Log-rank was used to investigate differences in Kaplan–Meier survival curves between groups. Survival was calculated from the day of the procedure. A survival census was last carried out on 1st of September 2025.

## 3. Results

During the study period, 33 patients underwent surgical LVR. The mean age was 62.1 ± 8.8 and 17 (51.5%) were females. Of these, 14 had sublobar or lobar A-LVRS (three bisegmentectomies, one basal segmentectomy, three extended segmentectomies and seven lobectomies, 42.4%) while the remaining 19 (57.6%) had the usual LVRS in the form of a long wedge resection through the target lobe.

The two groups were matched in all pre-interventional variables (Table 1), including age, gender, LFTs, CAT and mMRC dyspnea scores, 6MWT and others. However, there was a tendency for more RATS procedures in the A-LVRS group, while T-LVRS were performed mainly via a VATS approach (Table 1, RATS approach 78.6% vs. 47.4%, respectively, *p* = 0.064).

The duration of these procedures was similar and there were no intraoperative sequelae (Table 2). No conversions to thoracotomy were recorded, while the use of reinforced staplers was similar. Not all patients from both groups were admitted to the CCC unit post intervention and there were no patients needing an unplanned return to the CCC unit. The CCC-LOS was similar between the two groups, and although the LOS was not statistically different, it was shorter by 4 days in the A-LVRS group versus the T-LVRS group (8.4 vs. 13.1 days, *p* = 0.154). The DLS was shorter in the A-LVRS group than in the T-LVRS group (6.4 days versus 12.6 days, respectively, *p* = 0.042). Similarly, although similar numbers of patients were captured with complications in general, complications related to prolonged air leaks were captured more frequently in the T-LVRS rather than the A-LVRS group (57.9% versus 21.4%, respectively, *p* = 0.036). A larger volume of specimen was resected via the A-LVRS approach (1625 versus 352.4 cm^3^ for the T-LVRS group, *p* = 0.035).

The median follow-up was 18 months (3–60). Almost all patients in both groups reported an improvement in their breathing ability and overall quality of life (Table 3). FEV_1_ improved marginally more in the A-LVRS group (an increase of 8.25% versus 2.9% in the T-LVRS group, *p* = 0.049) and so did the TLCO (an increase of 7.9% versus a decrease of −1.01% in the T-LVRS group, *p* = 0.031). However, RV and 6MWT improved similarly in the two groups. CAT scores decreased more in the A-LVRS group than in the T-LVRS group (−17 versus −7.8, *p* = 0.019), but the mMRC did not.

**Figure 1:**
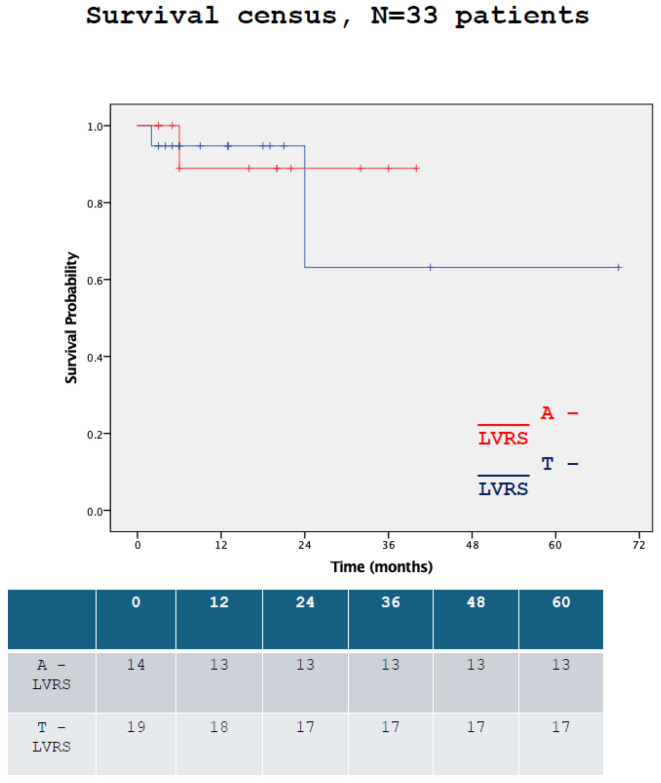
Survival by time between the two groups.

The in-hospital, 30-day and 90-day mortalities were similar between the two groups (Table 2). Survival until the end of the study was similar (log-rank chi-square = 0.487, *p* = 0.485, Figure 1).

## 4. Discussion

The main finding of this study was that A-LVRS showed modestly improved outcomes in some variables when compared with T-LVRS, measured 6–12 months post-intervention. Both types of LVRS were equally safe.

LVRS is traditionally performed in the form of a long wedge resection in order to avoid long and difficult dissections that would increase risks during surgery, especially in severe emphysema patients, who are by definition perceived as high-risk. Results from the present study show that sublobar or lobar anatomical resections did not develop into longer procedures, and there were no increased intraoperative adverse events (for example, bleeding); hence, such interventions can be safely performed when needed.

Additionally, in many instances of T-LVRS the stapling line is deployed onto emphysematous lung; theoretically, this could lead to increased air leaks, especially in cases where a whole lobe is destroyed by emphysema. Many times it is difficult to deploy a stapler on a non-emphysematous part of the target lobe without cutting the feeding vessels/bronchus. Results from this study show that T-LVRS resulted in longer drain stays and more issues related to air leaks than A-LVRS did, possibly because the stapler was deployed in such a way as to avoid diseased lung.

Air leaks remain the most frequent complication after LVRS, and are largely attributable to the fragile parenchyma. In the National Emphysema Treatment Trial, a significant percentage of patients experienced an air leak and nearly half developed a prolonged air leak (≥ 5–7 days), highlighting the vulnerability of staple-line closure in this setting [1,2]. By contrast, anatomical lung resections such as lobectomy or segmentectomy generally demonstrate lower rates of prolonged air leaks, reported as between 5% and 25% in large surgical series. These observations support the rationale that A-LVRS may decrease the incidence of air leak compared to traditional stapled resections, and this is supported by results from the present study.

Although a wedge LVRS is less technically demanding than a complex bisegmentectomy, the amount of lung resected with A-LVRS was larger than that of traditional LVRS. This finding is reported, to our knowledge at least, for the first time, and needs more investigation to determine its importance in the practice of LVR interventions in patients and its correlation to outcomes and breathing improvement. Interestingly, the magnitude of residual volume (RV) reduction following LVRS did not necessarily correlate directly with the amount of lung resected. This discrepancy can be explained by the physiological quality of the remaining parenchyma rather than the absolute resected volume. These findings underscore the importance of targeting resection based on the quality and recruitability of the remaining lung rather than the extent of volume loss alone [10].

An additional mechanism by which anatomical LVRS may enhance postoperative physiology relates to ventilation–perfusion matching. Traditional wedge LVRS leaves residual perfusion to adjacent, hyperinflated emphysematous units, whereas anatomical resections interrupt the bronchovascular pedicle of the target segment/lobe, effectively removing poorly ventilated tissue from both the ventilatory and perfusion circuits. Perfusion imaging after lung cancer surgery demonstrates greater redistribution of blood flow to preserved ipsilateral lobes after anatomical resections compared with limited resections, which is consistent with more efficient V/Q matching when vessels are divided. In parallel, LVRS reduces V/Q mismatch and improves gas exchange, and post-resection scintigraphy studies show shifts in ventilation and perfusion toward functioning lung. Together, these data suggest that anatomical LVRS could yield superior V/Q improvement—and therefore greater gains in spirometry—by eliminating perfusion to nonfunctional parenchyma [11].

Additionally, it could be hypothesized that as traditional wedge resection LVRS involves stapling through diseased and friable tissue, which may trigger a different localized inflammatory response compared to the clean anatomical planes of A-LVRS, this could possibly explain the difference in captured complications.

Few studies exist describing anatomical lung resections in severe emphysema patients conducted in patients with cancer. The present study involved emphysema patients taken through our service who underwent anatomical resection for this disease. The present study highlights the performance of anatomical lung resections in non-cancerous pathologies [12].

In this study, many anatomical lung resections were performed via the RATS approach, which could mean higher costs involved in those cases versus simple VATS wedge LVRS resections. More research, in terms of a full cost analysis, is warranted to clarify if improved outcomes, e.g., shorter LOS or DLS, would offset the extra costs incurred by using the RATS approach.

An important limitation of the present study is that it was a single-centre retrospective study involving a single surgeon’s practice. Also, the number of patients was limited, and hence larger scale studies are needed to ensure findings from this study are valid. Moreover, both groups were operated on with different minimally invasive approaches, i.e., robotic- or video-assisted thoracic surgery, and hence different outcomes could be attributed to this difference. Finally, no long-term tests are presented mainly because our service is fairly new, and as such the procedures of follow-up were yet to be determined, especially at the beginning of this study.

## 5. Conclusions

In conclusion, anatomical LVRS is safe, and in some compared outcomes it is better than traditional LVRS. However, it is not still clear if anatomical LVRS should be a substitute for the traditional LVRS technique.

## Figures and Tables

**Table 1:** Pre-interventional variables of N = 33 patients.

	T-LVRS (*n* = 19)	A-LVRS (*n* = 14)	*p*-Value
Age in years (mean ± SD)	61.2 ± 9.3	63.4 ± 8.1	0.485 *
Female gender (*n*, %)	7 (36.8)	10 (71.4)	0.107 **
BMI in Kg/m^2^ (mean ± SD)	25.7 ± 7.1	23.4 ± 5.3	0.324 *
FEV_1_ (mean ± SD)	39.6 ± 13.4	37.2 ± 12.7	0.618 *
TLCO (mean ± SD)	42.0 ± 14.7	41 ± 8.9	0.810 *
RV (mean ± SD)	220.7 ± 64.8	224.5 ± 56	0.865 *
CAT score (mean ± SD)	28.5 ± 4.1	31.1 ± 4.3	0.120 *
mMRC dyspnea (median, range)	4 (3–4)	4 (3–4)	0.998 ^$^
6MWT (mean ± SD)	210.8 ± 73.2	261.5 ± 89.8	0.187 *
AOT/LTOT (*n*, %)	7 (36.8)	9 (57.1)	0.114 **
Right-sided procedure (*n*, %)	7 (36.8)	4 (28.6)	0.424 ^‡^
Lower lobe target (*n*, %)	5 (26.3)	2 (14.3)	0.486 ^‡^
RATS approach (*n*, %)	9 (47.4)	11 (78.6)	0.064 **

LVR: lung volume reduction, T-LVRS: traditional lung volume reduction surgery, A-LVRS: anatomical resection lung volume reduction surgery, SD: standard deviation, BMI: body mass index, Kg: kilogram, m: meters, FEV_1_: forced expiratory volume in 1 sec, TLCO: transfer factor of the lung for carbon monoxide, RV: residual volume, CAT: COPD assessment test score, mMRC: modified Medical Research Council dyspnea score, 6MWT: 6 min walking test, AOT: ambulatory oxygen treatment, LTOT: long term oxygen treatment, RATS: robotic-assisted thoracoscopic surgery, *: Student’s *t*-test, **: chi-square test, ^$^: Mann–Whitney test, ^‡^: Fisher’s exact test.

**Table 2:** Comparison of surgical outcomes of N = 33 patients.

	T-LVRS (*n* = 19)	A-LVRS (*n* = 14)	*p*-Value
Duration of surgery in minutes (mean ± SD)	133 ± 67.1	145 ± 39.9	0.561 *
Intraoperative bleeding (*n*, %)	0 (0.0)	0 (0.0)	0.999 ^‡^
Conversion to thoracotomy (*n*, %)	0 (0.0)	0 (0.0)	0.999 ^‡^
Use of reinforced stapler (*n*, %)	10 (52.6)	5 (35.7)	0.272 ^‡^
Reoperation (*n*, %)	0 (0.0)	1 (7.1)	0.467 ^‡^
CCC postop (*n*, %)	10 (52.6)	9 (64.3)	0.745 **
Unplanned return to CCC postop (*n*, %)	0 (0.0)	0 (0.0)	0.999 ^‡^
CCC-LOS in days (mean ± SD)	2.58 ± 2.9	1.5 ± 1.2	0.323 *
DLS in days (mean ± SD)	12.6 ± 4.8	6.4 ± 4.5	0.042 *
LOS in days (mean ± SD)	13.1 ± 6.8	8.4 ± 6.3	0.154 *
Readmission to hospital (*n*, %)	4 (21.1)	1 (7.1)	0.095 ^‡^
Volume of resected specimen in cm^3^ (mean ± SD)	352.4 ± 229.1	1625.4 ± 1129.6	0.035 *
All complications (*n*, %)	11 (57.9)	10 (71.4)	0.486 ^‡^
Prolonged air leak related complications (*n*, %)	11 (57.9)	3 (21.4)	0.036 ^‡^
In-hospital/30-day mortality (*n*, %)	1 (5.3)	0 (0.0)	0.667 ^‡^
90-day mortality (*n*, %)	0 (0.0)	0 (0.0)	0.999 ^‡^

T-LVRS: traditional lung volume reduction surgery, A-LVRS: anatomical resection lung volume reduction surgery, SD: standard deviation, CCC-LOS: critical care complex length of stay, DLS: drain length of stay, LOS: length of stay, cm: centimeter, *: Student’s *t*-test, **: chi-square test, ^‡^: Fisher’s exact test.

**Table 3:** Comparison of outcomes of N = 33 patients 6–12 months post intervention.

	T-LVRS (*n* = 19)	A-LVRS (*n* = 14)	*p*-Value
FEV_1_ change % (mean ± SD)	2.9 ± 7.4	8.25 ± 5.5	0.049 *
TLCO change % (mean ± SD)	−1.01 ± 3.8	7.9 ± 2.4	0.031 *
RV decrease % (mean ± SD)	−54.7 ± 6.9	−59.0 ± 8.9	0.832 *
CAT score change (mean ± SD)	−7.8 ± 8.2	−17 ± 7.9	0.019 *
mMRC dyspnea change (median, range)	4 (3–4)	4 (2–4)	0.128 ^$^
6MWT change in meters (mean ± SD)	40.7 ± 8.3	47.5 ± 9.8	0.137 *
Subjective symptom/QoL improvement (*n*, %)	17 (89.5)	13 (92.9)	0.897 **

T-LVRS: traditional lung volume reduction surgery, A-LVRS: anatomical resection lung volume reduction surgery, SD: standard deviation, FEV_1_: forced expiratory volume in 1 sec, TLCO: transfer factor of the lung for carbon monoxide, RV: residual volume, CAT: COPD assessment test score, mMRC: modified Medical Research Council dyspnea score, 6MWT: 6 min walking test, QoL: quality of life, *: Student’s *t*-test, **: chi-square test, ^$^: Mann–Whitney test.

## Data Availability

Data presented in this study are available on request from the corresponding author.
